# Incorporating Augmented Reality Into Anatomy Education in a Contemporary Medical School Curriculum

**DOI:** 10.7759/cureus.57443

**Published:** 2024-04-02

**Authors:** Boris B Boyanovsky, Mostafa Belghasem, Brett A White, Samuel Kadavakollu

**Affiliations:** 1 Department of Biomedical Sciences, Rocky Vista University, Ivins, USA; 2 Department of Biomedical Sciences, Kaiser Permanente Bernard J. Tyson School of Medicine, Pasadena, USA; 3 Department of Clinical Science, Kaiser Permanente Bernard J. Tyson School of Medicine, Pasadena, USA; 4 Department of Biomedical Education, College of Osteopathic Medicine, California Health Sciences University, Clovis, USA

**Keywords:** anatomy assessment, hololens, cadaveric anatomy, medical education, virtual reality

## Abstract

Anatomy education in the medical school curriculum has encountered considerable challenges during the last decade. The exponential growth of medical science has necessitated a review of the classical ways to teach anatomy to shorten the time students spend dissecting, allowing them to acquire critical, new knowledge in other disciplines. Augmented and mixed reality technologies have developed tremendously during the last few years, offering a wide variety of possibilities to deliver anatomy education to medical students.

Here, we provide a methodology to develop, deliver, and assess an anatomy laboratory course using augmented reality applications. We suggest a novel approach, based on Microsoft^®^ HoloLens II, to develop systematic sequences of holograms to reproduce human dissection. The laboratory sessions are prepared before classes and include a series of holograms revealing sequential layers of the human body, isolated structures, or a combination of structures forming a system or a functional unit. The in-class activities are conducted either as one group of students (*n *= 8-9) with a leading facilitator or small groups of students (*n *= 4) with facilitators (*n *= 4) joining the groups for discussion. The same or different sessions may be used for the assessment of students’ knowledge.

Although currently in its infancy, the use of holograms will soon become a substantial part of medical education. Currently, several companies are offering a range of useful learning platforms, from anatomy education to patient encounters. By describing the holographic program at our institution, we hope to provide a roadmap for other institutions looking to implement a systematic approach to teaching anatomy through holographic dissection. This approach has several benefits, including a sequential 3D presentation of the human body with varying layers of dissection, demonstrations of facilitator-selected three-dimensional (3D) anatomical regions or specific body units, and the option for classroom or remote facilitation, with the ability for students to review each session individually.

## Introduction

Historically, cadaver dissection has been a substantial component of the medical school curriculum in the preclinical phases of student education [1-4]. Cadaver-based anatomy has traditionally been the first encounter of medical students with the inner workings of the human body and thus has been instrumental in educating future physicians not only about human structure but about the complexity of the human body with all its variations and pathology. Dissection has also illuminated students about the importance of humbleness and respect for the human body. However, the recent past has revealed several challenges to teaching anatomy in the classical method, necessitating a revision of the ways to teach this discipline for several reasons [5,6]. First, the tremendous advancement of the biomedical sciences and the volume of novel molecular mechanisms discovered, including the underlying biochemical and metabolic pathways, and particularly the exponential development of novel pharmacological treatment options, all of which require more space in the medical curriculum, thereby competing with the time allocated to the anatomical sciences [7,8]. Second, the advancement of modern, alternative approaches to anatomy education, including the creation of high-fidelity three-dimensional (3D) models and holographic platforms, has impacted anatomic education [3,5,6,9]. Finally, effective teaching approaches have been strained during the COVID-19 pandemic due to the inability to conduct in-person classes [10]. Although the pandemic has partially subsided, the awareness that similar possible events could occur remains, and medical educators should be prepared accordingly.

Given these considerations, combining the advantages of cadaveric dissection with a novel approach like augmented reality (AR) could be a solution to modernize anatomy education in contemporary medical school curricula [2,6]. Here, we offer a description of a novel approach to combine the advantages offered by Microsoft HoloLens AR technology using available anatomy platforms, such as Holo Human. The main goal of this paper is to provide anatomy facilitators with general guidelines on how to meaningfully incorporate AR into anatomy instruction in medical school curricula.

## Technical report

**Figure 1:**
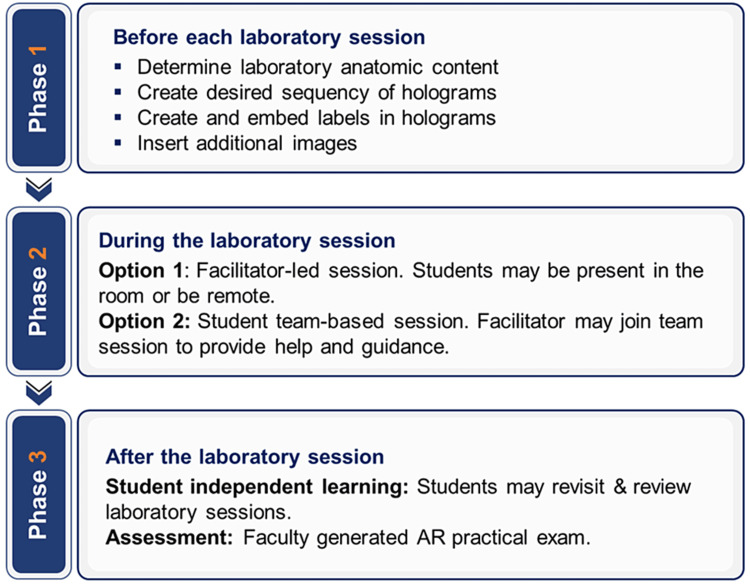
Flowchart describing the three phases of anatomy laboratory: design, in-session, and assessment. AR, augmented reality

Our proposed approach encompasses the design, delivery, and assessment of anatomy in system-based courses (Figure 1). We provide some insights into creating dissection-based anatomy labs using AR holograms, including several approaches to present anatomical material that provide advantages in visualizing the human body. We also provide an example of how to incorporate an AR-based presentation into a system-based course. Finally, we suggest ways to provide students the opportunity to use AR for independent learning and assessment of learned material. Our approach would be especially useful for students who are visual learners.

Phase 1: Approach to designing an AR-based anatomical laboratory

The first phase begins after the entire laboratory course is designed and the content of each laboratory session is assigned. Anatomists can adhere to the flow of curricular systems being taught without concern for sequence since AR allows flexibility to begin a dissection-based course with any region or system. This contrasts with the early phases of the classical, cadaveric approach to the anatomy curriculum, where starting with dissection of the back is necessitated for optimal handling of a real cadaver. In other words, this approach allows anatomists to better incorporate anatomy into a system-based curriculum with potential variations in the sequencing of the organ systems.

Compared to the classical cadaver-based dissection, AR also provides additional flexibility in selecting the sequence of the layers of dissection. Classical cadaver-based dissection always proceeds from more superficial layers to deep structures. AR allows following this sequence. Alternatively, it provides the opportunity to *build a body*. In other words, one can begin from the deepest structures, for example, the skeleton and gradually add overlying structures: muscles, vessels, and nerves. Finally, individual functional units or systems can be presented independently. We believe that the presentation of a single functional unit, for example, a muscle group and its innervation, creates a visual imprint and allows more reliable retention of the learned material.

AR also allows the introduction of clinical correlations during the sessions. This method allows students to discuss clinical cases with faculty in real time and relate the cases to the anatomic principles presented during the laboratory session. By embedding anatomic concepts into case-based clinical scenarios, medical students will likely have better long-term retention as well as a deeper understanding of the material [11].

Typically, the facilitator provides preparation material to students before each session. Our preference is a pre-recorded narrative, based on an MS PowerPoint slide deck, containing the material included in the laboratory. Alternatively, facilitators may choose a chapter or other sources.

Approach I

**Figure 2:**
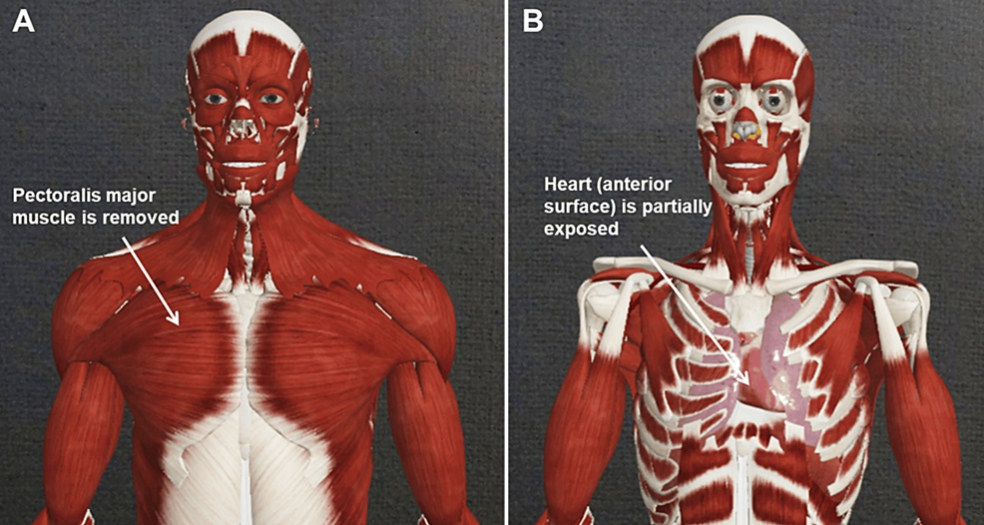
Example of dissection of the thorax, exposing the heart. All Images are reused with permissions from Complete Anatomy (www.3d4medical.com), copyright 3D4Medical Ltd., 2021.

The session designer prepares a specific set of holograms with the regions and structures the students will study. The first approach is to follow the canonic dissection from superficial to deep structures (Figure 2). We propose that the reader envision the *sequence of holograms* as a *slideshow* with dissection stages they want to present, only with 3D holograms instead of slides. Therefore, each hologram represents a separate layer of dissection. Here, a designer may manually remove structures during the laboratory session. (Here, we will not discuss the technical approach to create the desired content of each hologram.)

Approach II

**Figure 3:**
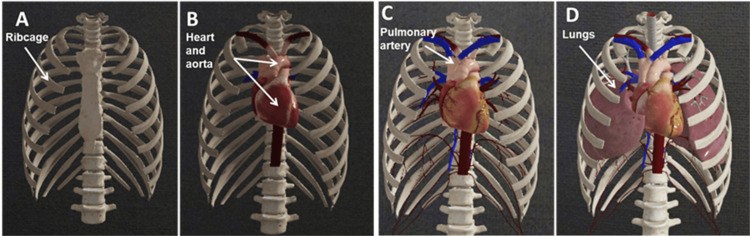
Example of adding thoracic structures to the thoracic cage. Image reused with permissions from Complete Anatomy (www.3d4medical.com), copyright 3D4Medical Ltd., 2021.

The second approach is to *build a body* by adding desired structures, beginning with deeper layers (Figure 3).

Approach III

**Figure 4:**
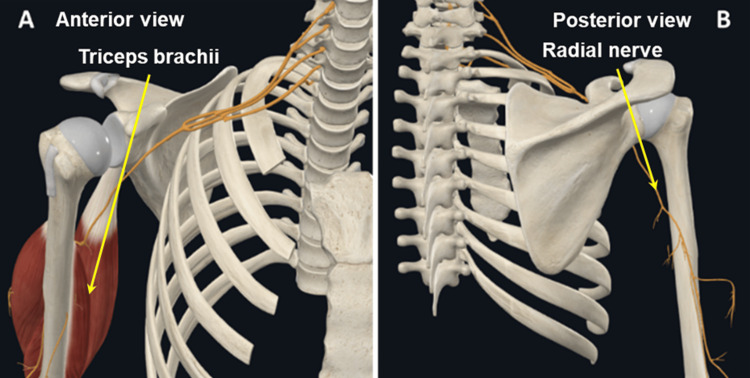
(A and B) Example of presenting a functional unit. In this case, the radial nerve innervates the triceps brachii muscle. The radial nerve can be traced up to its origin from the roots of the brachial plexus. Image reused with permissions from Complete Anatomy (www.3d4medical.com), copyright 3D4Medical Ltd., 2021.

The third approach is to construct a functional body unit, for example, a muscle group with attachment sites, innervation, and blood supply (Figure 4).

Also, during the preparation of the holographic sets, computed tomography (CT) scans or X-ray images can be shown simultaneously within the virtual reality field or separately, according to the possibilities provided by the anatomical platform used. Imaging may also be superimposed over holographic structures (Holo Anatomy, Case Western Reserve School of Medicine-provided platform) [4]. Some anatomy applications (Holo Human and Complete Anatomy, 3D4Medical) provide two-dimensional (2D) human anatomy atlases with life-size immersive holograms for collaborative learning, which may also be used along with the 3D holograms. In addition, other high-fidelity simulators provide an extension of their application, viewable through MS HoloLens headsets to support the learning of cardiac, abdominal, and OB-GYN ultrasound on one unique platform (CAE Vimedix).

Phase 2: Ways to present anatomical material in an AR-based laboratory

There are multiple approaches to delivering the prepared material. One is to have a leading facilitator who advances the holograms and explains the structures revealed. This approach allows the inclusion of a large audience and allows the facilitator to lead the laboratory, showing points of interest. Alternatively, the presenter may be in a remote location. This approach allows the invitation of outside specialists to discuss specific topics with a virtual demonstration. Another alternative approach is to create several small groups of students who will study the preset material at their own pace. The facilitators can join student groups for discussion or explanation as needed. Finally, the students may be gathered in the same room, or they may be situated remotely and still work together.

Phase 3 content: Access to sessions for student preparation and assessment

**Figure 5:**
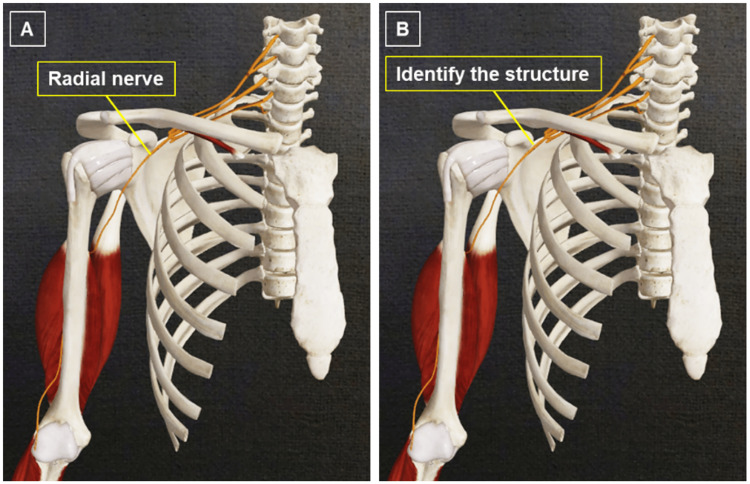
(A and B) Example of presenting a labeled and unlabeled structure for testing purposes. Image reused with permissions from Complete Anatomy (www.3d4medical.com), copyright 3D4Medical Ltd., 2021.

Phase 3 encompasses course assessment from the facilitators’ perspective and the learning of the material taught throughout the course from the student’s perspective [12]. After each lab or after the course, students continue to have access to the session material to work on and review. The advantages are numerous: the *dissection* may be accessed multiple times independently by multiple students, with the only requirement being access to an individual MS HoloLens headset. After the anatomy sessions of a system unit or a course, an assessment can be performed based on the material included in the anatomy laboratory sessions (Figure 5).

## Discussion

AR and mixed reality are developing and improving at a very fast rate, and their incorporation into numerous medical education modalities has been reported [11,13,14]. Simultaneously, anatomy is facing challenges in the constantly evolving biomedical field. High-fidelity, 3D holograms are being used to prepare students for future encounters with AR-based learning and practice [14,15].

Several schools have started incorporating HoloLens-based anatomy platforms, either to completely replace cadavers or to do it along with some form of cadaver dissection programs. These approaches have shown equal effectiveness in learning compared to using cadavers alone, as well as a reduction in the amount of time students spend dissecting and learning [6,13,15]. The inclusion of AR in the teaching process also helps satisfy the needs of *visual learners*.

We believe that our novel approach may help anatomists design, deliver, and assess anatomy with the help of AR holograms. The novelty of our approach is the inclusion of a variety of preset sequences of holograms to deliver anatomy holistically. Additionally, enhancing the *visibility* of complex 3D structures of the human body helps students to better understand and retain knowledge while saving time and avoiding suboptimal dissection. Our model can be used as a stand-alone or in combination with other modalities, such as cadavers (pro-section) or plastinated models.

The Microsoft HoloLens headset offers distinct benefits of AR and mixed reality over virtual reality. These include the removal of direct cabling between the computer and the headset and the presentation of a 3D hologram picture overlaid and interacting with the viewer within a real physical environment. The MS HoloLens headset experience does not replace the interaction with a real human cadaver for a first-year medical student, but it allows the student to interact with the exact 3D image of the human body an unlimited number of times at an early stage of their journey in medical learning.

Challenges

AR is not without challenges, as with any other teaching modality. From a didactic standpoint, the most prominent challenge is the relative lack of realism of the holograms to cadavers. This is partially compensated for by the fact that they are created to resemble *live* structures. However, this is also not a *perfect* match to a real, live structure, as would be seen during surgery, for example.

The second is technical challenges. As of now, a few of the available anatomical platforms are designed to help the anatomist prepare their own sessions. Most platforms provide software with premade settings, which are helpful per se, but do not always fulfill the specific structure-unit configuration necessary for the anatomist in each session. Therefore, a considerable amount of work is required in Phase 1. However, the possibility of saving these sessions for future use makes the investment worthwhile.

The third challenge is the learning style of students, which affects study time [16]. Although some students would prefer the direct touch with cadavers, which is an invaluable learning experience, we believe that AR can significantly contribute to helping students understand the 3D relations in humans and can be a valuable addition to cadaveric dissection or *stand-alone* learning method [16]. 

Finally, after prolonged usage, we have observed that some eye strain occurs. To avoid this undesired effect, we recommend switching from a HoloLens presentation to some discussion - either clinical cases or imaging modalities - to supplement the session.

Future directions

With the exponential growth and development of AR and the increasing incorporation of this technology into the field of medicine, we anticipate that pathology, histology, and functional aspects of the human body will soon be developed and integrated into the technology. In addition, there is an increasing reliance on robots in medical fields such as surgery, which will likely also incorporate AR to a greater degree.

Moving forward, we plan to work on combining plastinated models into holographic imaging through 3D scanning. This approach has its own merits. For example, students will study the models in the lab and then at home using the hologram.

## Conclusions

We do not compare a cadaver-based laboratory with an AR-based laboratory in this paper. In addition, we understand that each method has its unique possibilities, which can certainly be used in combination. Yet, we have hopefully provided a roadmap for other institutions looking to implement AR methods into their medical school curricula.
